# Nervous and Mental Diseases

**Published:** 1906-03

**Authors:** R. F. Sheehan

**Affiliations:** Buffalo, N. Y.; Late House Physician at the Buffalo German Hospital


### PROGRESS IN MEDICAL SCIENCE.

### Nervous and Mental Diseases

By R. F. SHEEHAN, M. D., Buffalo, N. Y.
Late House Physician at the Buffalo German Hospital,

Therapeutic Notes upon Veronal in the Insane by Serieux and Mignot (*Archives de Neurologie,* 109, V. XIX '05) who record the result of careful observation upon twenty cases. They are not picked cases, but were mostly old men and women. The drug was given with a hot drink, or in suspension in milk or water or even mixed with food.

MELANCHOLIA—Its action was quite remarkable. In doses of 0.30 to 0.50 it was superior to all other hypnotics. Sleep came in one to two hours and was usually calm and sufficiently long. In spite of long continuance there was no habituation. In addition to the excellent hypnotic effect, it produced a dimunition in the agitation and anxiety, but was without influence upon the delirium or depression.

DEMENTIA PRECOX—It was equally satisfactory here, except in one catatonic case.

PARESIS—Here the action was less satisfactory, inferior to the bromides, and prolonged baths. Sleep came on only after four or five hours and was of short duration.

CONCLUSIONS. 1. In melancholia and agitated dementia, veronal is an excellent hypnotic. Sleep comes on usually in from one to two hours and lasts several hours. Its action is more marked after several days usage. There is no habituation and it does not lose its good effect easily. The dose is from 0.3 to 1.0, never above the latter mark. It exerts as well, a decided calmative effect in this class of psychoses.

2. Its action in general paralysis (agitated and hallucinatory forms) is not satisfactory. Sleep comes slowly, is irregular and of short duration. In depressed and simple and demented forms of paresis the authors have not had sufficient experience.

3. Even in doses of 1.0 they never found disagreeable symptoms which could be attributed to the drug. There was no modification of pulse, no alteration of cardiac murmurs; old people, even those with cardiac lesions, bore the drug well. Albumenuria was never observed.

4. In only one case out of forty was there a marked intolerance. The drug was often given for long periods night after night. They regard it as the most serviceable of hypnotics.—*Lancet Clinic.*

